# Electrospun PLGA Fiber Diameter and Alignment of Tendon Biomimetic Fleece Potentiate Tenogenic Differentiation and Immunomodulatory Function of Amniotic Epithelial Stem Cells

**DOI:** 10.3390/cells9051207

**Published:** 2020-05-13

**Authors:** Mohammad El Khatib, Annunziata Mauro, Miriam Di Mattia, Ralf Wyrwa, Martina Schweder, Massimo Ancora, Francesco Lazzaro, Paolo Berardinelli, Luca Valbonetti, Oriana Di Giacinto, Andrea Polci, Cesare Cammà, Matthias Schnabelrauch, Barbara Barboni, Valentina Russo

**Affiliations:** 1 Unit of Basic and Applied Biosciences, Faculty of Bioscience and Agro-Food and Environmental Technology, University of Teramo, 64100 Teramo, Italy; melkhatib@unite.it (M.E.K.); mdimattia@unite.it (M.D.M.); pberardinelli@unite.it (P.B.); lvalbonetti@unite.it (L.V.); odigiacinto@unite.it (O.D.G.); bbarboni@unite.it (B.B.); vrusso@unite.it (V.R.); 2 Department of Biomaterials, INNOVENT e. V., 07745 Jena, Germany; rw1@innovent-jena.de (R.W.); ms@innovent-jena.de (M.S.); 3 Department of Surface Engineering, INNOVENT e. V., 07745 Jena, Germany; ms1@innovent-jena.de; 4 Laboratory of Molecular Biology and Genomic, Istituto Zooprofilattico Sperimentale dell’Abruzzo e del Molise “Giuseppe Caporale, 64100 Teramo, Italy; m.ancora@izs.it (M.A.); c.camma@izs.it (C.C.); 5 Research & Development Department, Assut Europe S.p.A., Magliano dei Marsi, 67062 L’Aquila, Italy; francesco.lazzaro@assuteurope.com; 6 Laboratory of Diagnosis and surveillance of foreign diseases, Istituto Zooprofilattico Sperimentale dell’Abruzzo e del Molise “Giuseppe Caporale, 64100 Teramo, Italy; a.polci@izs.it

**Keywords:** tendon, amniotic epithelial stem cells, fiber diameter size, aligned fibers, tendon tissue engineering, PLGA, electrospinning, biomimetic scaffold, tenogenic differentiation, immunomodulation

## Abstract

Injured tendons are challenging in their regeneration; thus, tissue engineering represents a promising solution. This research tests the hypothesis that the response of amniotic epithelial stem cells (AECs) can be modulated by fiber diameter size of tendon biomimetic fleeces. Particularly, the effect of electrospun poly(lactide-co-glycolide) (PLGA) fleeces with highly aligned microfibers possessing two different diameter sizes (1.27 and 2.5 µm: ha1- and ha2-PLGA, respectively) was tested on the ability of AECs to differentiate towards the tenogenic lineage by analyzing tendon related markers (Collagen type I: COL1 protein and mRNA Scleraxis: *SCX*, Tenomodulin: *TNMD* and *COL1* gene expressions) and to modulate their immunomodulatory properties by investigating the pro- (IL-6 and IL-12) and anti- (IL-4 and IL-10) inflammatory cytokines. It was observed that fiber alignment and not fiber size influenced cell morphology determining the morphological change of AECs from cuboidal to fusiform tenocyte-like shape. Instead, fleece mechanical properties, cell proliferation, tenogenic differentiation, and immunomodulation were regulated by changing the ha-PLGA microfiber diameter size. Specifically, higher DNA quantity and better penetration within the fleece were found on ha2-PLGA, while ha1-PLGA fleeces with small fiber diameter size had better mechanical features and were more effective on AECs trans-differentiation towards the tenogenic lineage by significantly translating more efficiently SCX into the downstream effector TNMD. Moreover, the fiber diameter of 1.27 µm induced higher expression of pro-regenerative, anti-inflammatory interleukins mRNA expression (IL-4 and IL-10) with favorable IL-12/IL-10 ratio with respect to the fiber diameter of 2.5 µm. The obtained results demonstrate that fiber diameter is a key factor to be considered when designing tendon biomimetic fleece for tissue repair and provide new insights into the importance of controlling matrix parameters in enhancing cell differentiation and immunomodulation either for the cells functionalized within or for the transplanted host tissue.

## 1. Introduction

Tendinopathies, common musculoskeletal pathologies, represent about 30% of tendon-related injuries [1,2,3]. Around 30 million tendon interventions take place in human medicine [4,5] with worldwide increasing prevalence. Although healing can occur, tendon injuries remain difficult to manage and often lead to the formation of scar tissue instead of the organized extracellular matrix (ECM) possessing inferior biochemical properties with altered functionality especially in terms of movement and strength. This can be devastating for the daily life of patients as it may dramatically impose a reduction in mobility associated with chronic pain [6] accompanied by increasing the chances of recurrences and tendon ruptures [1,7,8]. Current treatments for tendinopathies, including the use of conservative approaches, such as rest, medication (non-steroidal, anti-inflammatory or corticosteroid drugs), cryotherapy, or surgical repair using auto-, allo-, and xeno-grafts, have all shown limited success [9]. Innovative strategies that can regenerate tendons and improve their biomechanical properties are therefore urgently sought.

A promising approach is the interdisciplinary field of tissue engineering aiming to create tissue-like structures by proper combination of several elements like cells, biological or/and biomaterials with biochemical and physical signals [10].

Even if there has been a significant improvement in tendon tissue engineering, the ideal stem cell source remains still to be defined. Amongst the stem cells used, multipotent mesenchymal stem cells (MSCs) stand out mainly for their self-renewal, differentiating, and immunomodulatory abilities, making them an important cell source for possible application in tendon regeneration. Different MSCs types have been applied and analyzed for their in vitro and in vivo tendon healing such as tendon stem/progenitor cells (TSPCs) [11,12,13], bone marrow mesenchymal stem cells (BMMSCs) [14,15,16], and adipose-tissue derived mesenchymal stem cells (ADMSCs) [17,18,19,20]. However, some studies have reported that the use of MSCs may form ectopic bone and express alkaline phosphate (ALP) after being implanted in tendons in vivo [21,22]. In tendons and ligaments, TPSCs have been demonstrated [11], but are unsatisfactory in sustaining tendon healing in adults [23]. Moreover, their isolation and collection are very difficult for donor site morbidity [23]. ADMSCs have a potential therapeutic effect as demonstrated by several studies. This stem cell source can modulate the tendon microenvironment in vitro and to improve tendon regeneration in vivo. However, further researches will be necessary to clarify the mechanisms behind these biological responses [24].

Amniotic epithelial stem cells (AECs) have been recently identified as an alternative and promising cell source in tendon regenerative medicine. These cells have shown a multilineage differentiation potential in vitro [25], non-tumorigenicity [26], low immunogenic profile [27,28], and anti-inflammatory and immunomodulatory properties [25,29,30,31,32], which make them an ideal candidate for regenerative medicine and tissue repair. Indeed, these types of cells possess the innate ability to modulate maternal-fetal immune-mediated processes [33]. Additionally, it has been demonstrated AECs’ ability to differentiate towards the tenogenic lineage both in vitro and in vivo together with their regenerating potential [29,34,35,36,37]. 

Extensive injuries, such as tendon ruptures, cannot be healed by cell-based therapy only, and require the use of specific biomaterial scaffold. Based on the knowledge about native tendon tissue complexity, a biomimetic approach was followed as the design criteria for the fabrication of an “ideal” scaffold. Considering the morphology of native tendons and their oriented fibrillary structure, the electrospinning process was selected for its capability to obtain fibrous constructs, with an average diameter ranging between the nano- and the microscale, resembling the architecture of the native tendon ECM [38].

Poly-(l-lactide-co-glycolide) (PLGA) is a versatile FDA approved co-polyester that has gained considerable attention in many clinical applications such as suture anchors, fracture fixation, orthopedic implants, drug delivery, and tendon/ligaments reconstruction [39,40,41,42,43,44,45]. Moreover, PLGA has been widely used for tendon tissue engineering where it has been engineered with different cell sources [46,47,48,49], amongst which AECs [50,51].

Recently, it has been shown that this alignment of the fibers can induce a tenogenic commitment of the seeded stem cells [51,52,53]. Those electrospun highly aligned PLGA fleeces mimic collagen fibers of the tendon ECM and can induce an early epithelial-mesenchymal transition (EMT) and tenogenic differentiation on AECs [51]. This type of stem cells allows one to verify the topological effect of the scaffolds in depth and to study the mechanisms of an epithelial cell, cuboidal in its morphology, which normally does not express collagen type I, to differentiate towards the mesenchymal tenogenic lineage.

Furthermore, the process parameters for electrospinning can be tuned not only to fabricate aligned fibers but also to control their diameter size that in turn affects cell behavior [54,55,56]. Indeed, it has been reported that the response of a variety of cell types including fibroblasts [46,57], and MSCs [48,58] can be modulated by fiber alignment and diameter size. Cultivating tendon fibroblasts on aligned PLGA meshes, Eriksen et al. observed a fiber diameter-dependent response, through which nanofibers regulated ECM production and cell proliferation, while the microfibers upregulated fibroblastic expression markers [59]. Accordingly, Lee et al. demonstrated that nanoscale fibers of the early-stage repair model elicit the proliferative phase of wound repair, while the micron-sized fibers of the mature model were more representative of the remodeling phase involving ECM alignment as a crucial factor for avoiding scar formation and promoting healing of soft tissue injuries [60].

With these ‘‘immunoregenerative’’ biomaterial strategies, optimal material geometries in the micro- and nano-scale, can be created that greatly impact material–host interactions and best regulate host inflammatory response [61]. In this context, the immunoregulatory function of MSCs cultured on aligned fibers scaffold was enhanced compared to MSCs cultured on randomly arranged fibers [62], and the secretion of proinflammatory molecules by macrophage cells (Mφ) was mainly dependent on fiber diameter [63]. Therefore, it would be of great benefit to design scaffolds capable of boosting stem cell immunomodulatory functions to avoid aberrant immune responses and contribute to efficacious tissue regeneration. 

According to these premises, the main objective of this study was to design electrospun PLGA fleeces for tendon regeneration from highly oriented fibers with two different diameters (about 1.27 and 2.5 µm), which, following a biomimetic approach, are in the range of collagen fibers [64,65]. On these PLGA fleeces, it was evaluated the effects of fiber diameter size on the biology of AECs, especially in terms of their teno-differentiation and immunomodulation capacity, in the absence of biological factor supplementation.

## 2. Materials and Methods

### 2.1. Ethical Statement

No ethical statement is required since the amniotic membranes used in the present research were collected from waste reproductive tissues of animals slaughtered for food purposes.

### 2.2. Materials

Poly(lactide-co-glycolide) (PLGA, PLG8523) with an inherent viscosity midpoint of 2.3 dL/g and a lactide to glycolide ratio of 85:15 was purchased from PURASORB^®^ (Corbion Purac, Gorinchem, The Netherlands). The molecular weight was determined by gel permeation chromatography (GPC) in chloroform with polystyrene as the external standard to be M_w_ = 258,000 g/mol. Hexafluoro-2-propanol (HFIP) was obtained from Apollo Scientific, Manchester, UK with a purity of 99%. All other chemicals and solvent were of analytical grade and used as received.

### 2.3. Synthesis of The Aligned PLGA Microfiber Fleeces by Electrospinning Technique

Electrospun PLGA fibers of varying diameters were fabricated using a commercial E-Spintronic electrospinning apparatus (Erich Huber, Gerlinden, Germany) with climate control. Briefly, 8% and 12% (%*wt*/*wt*) of PLGA were prepared by dissolving the weighed masses in hexafluoroisopropanol (HFIP) under magnetic stirring overnight. Electrospinning was performed using a 5 mL polypropylene syringe set on the pump and connected to the needle (inner diameter 0.4 and 0.8 mm for PLGA 8% and 12% (%*wt*/*wt*, respectively), respectively with a 35 cm PTFE tube (Intra Special Catheters, Rehlingen-Siersburg, Germany). The parameters controlling the resulted fiber fleeces were varied, as shown in Table 1, to fabricate PLGA fibers with different diameters. Electrospun PLGA fleeces were collected on aluminum foil placed onto the metallic cylindrical drum rotating collector, with a diameter of 12 cm (related to a circumference of about 38 cm). The rotational speed of the collector was set at 1000 rpm to produce PLGA fleeces with highly aligned fiber topography (ha-PLGA). In this study, fleeces with diameters averaging 1.27 ± 0.11 µm and 2.5 ± 0.27 µm (ha1-PLGA and ha2-PLGA, respectively) were fabricated by electrospinning 250 µL of the PLGA solution.

### 2.4. Characterization of The Microfiber PLGA Fleeces

#### 2.4.1. Scanning Electron Microscopy (SEM)

The microscopic morphology of the fabricated PLGA fleeces has been assessed by scanning electron microscopy (SEM). The fleeces were air- and vacuum-dried, and their surfaces were gold-coated using a sputter coater to ensure the electrical conductivity of the analysis. The specimens were then imaged using a Supra 55VP field-emission SEM at an accelerating voltage of 5 kV (Carl Zeiss AG, Jena, Germany). The angle distribution, divided from −45° to +45°, the pore size distribution, as well as the average fiber diameter size measured from around 100 fibers, randomly chosen, from each fleece type (n = 3 for each fleece type), were assessed using ImageJ software (NIH image).

#### 2.4.2. Fourier Transform Infrared Spectroscopy (FT-IR) Analysis

The FT-IR spectra of the two PLGA fleeces have been characterized before and after sterilization using an FT-IR spectrometer Nicolet iS10 (Thermo Fisher Scientific S.p.A., Milan, Italy). Sixty-four accumulations with a resolution of 4 cm^−1^ operating in the range of 4000–650 cm^−1^ have been used to collect the average spectra. Three different locations from three samples of each type were analyzed. 

#### 2.4.3. Fleece Mechanical Tests

The tensile properties of the electrospun fibrous mats were determined using a material testing machine (ACQUATI, Acquati Giuseppe, Italy) equipped with a 10N load cell. Rectangular shaped samples (dimensions: 30 mm × 5 mm, n = 5 each type of fleece) in the dry state were stretched at a constant crosshead speed of 10 mm/min along the direction of the highly aligned fibers. The cross-sectional area (width and thickness) for each sample was calculated to determine the structural properties of the tested fleeces. Ultimate tensile strength and fracture strain were computed from the generated stress-strain curves and the structural properties of the fleeces were represented by the maximum load (N), Ultimate Tensile Strength (UTS, MPa), fracture strain (%), and Young’s modulus (MPa).

### 2.5. PLGA Fleeces Sterilization

Before cell seeding, electrospun PLGA fleeces with the two different diameter size were cut into rectangular pieces 10 × 5 mm, sterilized with 70% ethanol (EtOH) in 0.9% NaCl/distilled water (diH_2_O) for 10 s followed by washing with sterile phosphate-buffered saline (PBS; Sigma Chemical Co. St. Louis, MO, USA) for 10 min as previously reported by Russo et al. [50]. The fleeces were then conditioned in cell culture growth medium (GM) composed by Minimum Essential Medium Eagle-α modification (α-MEM) supplemented with 10% Fetal Bovine Serum (FBS), 1% Ultra-Glutamine, 1% Amphotericin B, 1% Penicillin/Streptomycin, and incubated at 38 °C with 5% CO_2_ for 24 h.

### 2.6. Isolation of Ovine AECs

Amniotic membranes were obtained from the discarded placenta of slaughtered sheep of Appenninica breed at approximately 2–3 months of pregnancy; and ovine AECs (oAECs) were isolated, as detailed previously in Barboni et al. [36], from the amniotic membranes of at least three fetuses. Briefly, the amniotic membrane was washed with PBS and cut aseptically into small pieces from which oAECs were isolated from its epithelial layer after enzymatic digestion (0.25% Trypsin-EDTA, Sigma Chemical, St. Louis, MO, USA) as previously described [36]. Cell suspension was filtered through a 40 µm cell filter, into a 50 mL falcon tube containing 20% FBS to inactivate Trypsin. After centrifugation, cells were counted with vital Trypan-Blue stain by using a hemocytometer. The oAECs, at a concentration of 3 × 10^3^ cells/cm^2^, were cultured in GM and incubated at 38 °C with 5% CO_2_. Reached 70% of confluence, cells were detached with 0.25% Trypsin-EDTA (Sigma Chemical, St. Louis, MO, USA) and cultured in GM at the same concentration. The oAECs, before their use, were characterized by flow cytometry to confirm their negativity for hemopoietic markers (CD14, CD58, CD31, and CD45), positivity for surface adhesion molecules (CD29, CD49f, and CD166) and stemness markers (TERT, SOX2, OCT4, and NANOG), a low expression for MHC class I molecules, and the absence of MHC class II (HLA-DR) antigens [29,34,36], and their negativity for *SCX*, *COL1* and *TNMD* gene expression [34].

For the experimental condition, oAECs (0.05 × 10^6^) were then cultured on Petri dishes or seeded onto sterilized PLGA fleeces (ha1-PLGA and ha2-PLGA) in the presence of GM (supplemented with 10% FBS) and incubated at 38 °C with 5% CO_2_ for different time periods (4, 24, and 48 h, or 7 days, depending on the analysis described below).

### 2.7. DNA Extraction and Quantification

Total genomic DNA was extracted from 0.05 × 10^6^ oAECs before seeding, cultured on Petri dishes and seeded onto both types of PLGA fleeces at 4 h and 48 h of culture (n = 3 of sample/time point) by Maxwell 16 cell DNA purification kit, according to the manufacturer’s instructions (Promega, Madison, WI, USA).

All samples were analyzed using a fluorescence-based DNA quantification approach by fluorescent nucleic acid binding dyes. The Qubit Quantitation Platform calculates concentration based on the fluorescence of the Qubit^®^ dsDNA HS Assay (Life Technologies^TM^), which binds to double-stranded DNA.

### 2.8. AECs’ Viability on Fleeces

Ovine AECs seeded onto electrospun ha1- and ha2-PLGA fleeces were evaluated for their survival after 24 h, 48 h and 7 days culture (n = 3 for each type of fleece/time point). To determine cell viability, cells were stained for 30 min with calcein AM (vital green fluorescent dye, 4 µM) and with propidium iodide (PI red fluorescent dye, 12 µM) to detect non-viable cells. Axioskop 2 Plus incident light fluorescence microscope (Carl Zeiss, Oberkochen, Germany) equipped with a CCD camera (Axiovision Cam, Carl Zeiss) with a resolution of 1300 × 1030 pixels, configured for fluorescence microscopy, and interfaced to a computer workstation, provided with an interactive and automatic image analyzer (Axiovision, Carl Zeiss) was used for images acquisition. The viable and dead cell numbers were counted as positive green (calcein AM) or red (PI) cells/ 100 cells, respectively. For nuclear counterstaining, Hoechst 3342 was used at the final dilution of 1:2000 for 15 min at RT. 

### 2.9. Spatial Distribution, Penetration, and Morphology of oAECs on the Seeded PLGA Fleeces

AECs on Petri dishes, used as control (oAECs), or onto electrospun ha1-PLGA and ha2-PLGA fleeces were immuno-stained with F-actin filament stain (phalloidin) to investigate cell distribution, penetration and cytoskeleton after 24 h and 48 h culture (n = 3 for each type of fleece/analysis/time point). In detail, after culture, cells were fixed in 4% paraformaldehyde (10 min) and permeabilized with PBS + Triton X-100 (0.1%) for 10 min at room temperature (RT). After washing with PBS, phalloidin-TRITC (1:10) (Sigma Aldrich, Missouri, USA) in PBS was added to each sample (20 min) followed, for nuclear counterstaining, by DAPI (Vectastain) in PBS used at the final dilution of 1:5000 for 15 min at RT. Samples were observed under a Nikon Ar1 laser confocal scanning microscope (Nikon, Düsseldorf, Germany) equipped with the NIS-Element software, using a Plan Apo λ 40X oil objective (numerical aperture 1.3; zoom 1.00X; Refractive Index: 1.515; pinhole size: 12.8 µm; pixel size; 0.63 µm; 1 picture every 0.15 s). The used channels were as follow:

Channel 1: DAPI; λ_exc_ = 404 nm; λ_em_ = 450/50 nm, at 81% of the maximum laser power.

Channel 2: TRITC; λ_exc_ = 561.5 nm; λ_em_ = 595/50 nm at 0.6% of the maximum laser power.

Changes in cell morphology were assessed among the different samples by measuring the nuclei length and the elliptical form factor to distinguish between ovoid and elongated cell as described previously [66]. The ratio of length to breadth of the object (cell) was considered as the elliptical form factor. Cells with a length >30 and elliptical factor form >1.7 were considered elongated while those for ovoid cells were length <25 and elliptical form factor <1.5. The cellular aspect ratio was calculated by dividing the cell width by its length. The lower is the ratio, the more is the cell elongated. Three replicates were considered for each experiment.

Additionally, after acquiring XZ projection from confocal microscopy Z-stacks at a magnification of 40×, a color gradient to the pixels with the highest intensity values of the Z-sequence was generated on the images using the depth-coded MaxIP (Maximum Intensity Projection) option to determine cell penetration property of the cells cultured onto either ha1- or ha2-PLGA fleeces [51].

### 2.10. Evaluation of Tenogenic Differentiation on oAECs Seeded on PLGA Fleeces

#### 2.10.1. Immunofluorescence for Collagen Type I

Immunofluorescence technique was used to assess the Collagen type I (COL1) protein expression in oAECs seeded on Petri dishes as well as onto both ha1-PLGA and ha2-PLGA fleeces was assessed after 24 h and 48 h culture (n = 3 for each type of fleece/analysis/time point). In detail, the samples were fixed in 4% paraformaldehyde/PBS (10 min) and permeabilized in 0.05% Tween 20/1% BSA/PBS for 10 min at RT. After washing with PBS, non-specific binding was blocked, incubating the samples with 1% BSA/PBS for 1h at RT followed by incubation with the COL1 primary antibody (EMD Millipore Corporation, Temecula, USA) diluted in PBS (1:100), overnight at 4 °C. Finally, cells were exposed to anti-Mouse Cy3 conjugated secondary antibody (Sigma-Aldrich, St. Louis, MO, USA) diluted in PBS (1:500) for 40 min at RT. Nuclear counterstaining was obtained with DAPI (Vectastain) in PBS used at the final dilution of 1:5000 for 15 min at RT. In all experiments, non-immune serum was used in place of the primary antisera as a negative control. All controls performed were negative. Samples were analyzed using an Axioskop 2 Plus incident light fluorescence microscope (Carl Zeiss) equipped with a CCD camera (Axiovision Cam, Carl Zeiss), as described above.

#### 2.10.2. Tendon-Related Gene Expression Profile by Real Time RT-qPCR

The reverse transcriptase quantitative real-time polymerase chain reaction method (RT-qPCR) was performed to compare the mRNA expression of tenogenic [35] and immunomodulatory related genes [67] in oAECs cells alone and in oAEC seeded on different PLGA fleeces (ha1-PGLA and ha2-PLGA) at 24 h, 48 h for the tendon related genes and at 24 h, 48 h and 7 days of culture for the immuno related genes (n = 3 for each type of sample/time point). Total RNA was extracted by using TRIzol (Sigma), according to the manufacturer’s instructions. Integrity and size of extracted total RNA were evaluated by 1% agarose gel electrophoresis using GelRed staining (Bioline). Quantification of total RNA samples was evaluated by using Thermo Scientific NanoDrop 2000c UV-Vis spectrophotometer at 260 nm. Digestion of genomic DNA, carried out by using DNaseI (Sigma) for 15 min at RT, was performed before reverse transcription (RT). Then, 1 μg of total RNA of each sample was used for cDNA synthesis using RT reaction with Random Hexamers primer and Tetro Reverse Transcriptase (Bioline) at a final volume of 20 μL, according to the manufacturer instructions. Real-time qPCR analysis was performed by using SensiFAST^TM^ SYBR Lo-ROX kit (Bioline), according to the manufacturer instructions, using genes’ primer sequences for *SCX*, *COL1*, *TNMD* tenogenic related, pro-inflammatory interleukins 6 (IL-6) and 12 (IL-12b) and anti-inflammatory interleukins 4 (IL-4) and 10 (IL-10) related genes (see Table S1). The reaction was carried out by using the two-step cycling protocol for 40 cycles (10 s at 95 °C for denaturation and 30 s at 60 °C for annealing/extension) with 7500 Fast Real-time PCR System (Life Technologies) followed by melt-profile analysis (7500 Software v2.3). For each gene expression, each sample was performed in triplicate, and values were normalized to GAPDH endogenous reference gene (Table S1). The relative gene expression was calculated by the comparative Ct (ΔΔCt) method and converted to relative expression ratio (2^−ΔΔCt^) [68]. For primers details see Table S1.

### 2.11. Statistical Analysis

The analyses have been carried out on oAECs of at least 3 fetuses (n = 3 biological replicates) by analyzing quantitative data of each sample in triplicate for each analysis expressed as mean ± Standard Deviation (S.D.). The results were assessed for normal distribution using D’Agostino and Pearson tests. For biological analysis, One-way ANOVA multi-comparison tests followed by Tukey post hoc tests were used for data sets comparison by using GraphPad Prism 8 (GraphPad Software, San Diego, CA, USA). Statistical analysis for assessing the ultrastructure and mechanical properties of both PLGA fleeces was performed by two-tailed independent *t*-test (GraphPad Prism 8, GraphPad Software, San Diego, CA, USA). The values were considered statistically significant for at least *p* < 0.05.

## 3. Results

### 3.1. Morphology and Characterization of the Microfiber PLGA.

**Figure 1:**
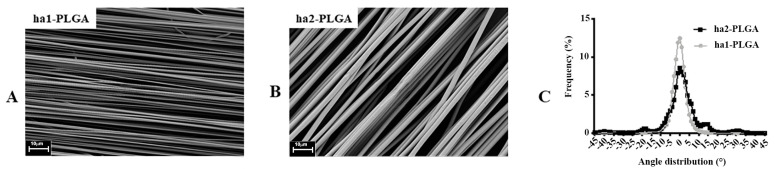
Morphological characteristics of the electrospun PLGA microfibers. (**A**,**B**) SEM micrographs of PLGA with 1.27 ± 0.11 µm (ha1-PLGA) and 2.5 ± 0.27 µm (ha2-PLGA) highly aligned fibers showing defect-free fleeces (n = 3 for each type of fleece). Scale bars = 10 µm. (**C**) Frequency distribution of fiber orientation within ha1- and ha2-PLGA fleeces. Fibers of both PLGA fleeces show a sharp Gaussian curve with an orientation angle mainly ranged between −10° to +10° with a statistically significant high fiber alignment in the case of the fibers with small fiber diameter size (ha1-PLGA, *p* < 0.05) (n = 3 for each type of fleece).

After a series of optimization of the electrospinning process parameters, two PLGA fleeces with highly aligned fibers (ha1-PLGA and ha2-PLGA) possessing two different diameter sizes have been chosen for further investigations in this study in which the fabrication conditions are reported in Table 1. SEM investigations of the selected electrospun ha1-PLGA and ha2-PLGA fleeces show a high degree of alignment, reproducible, as well as defect-free structures (Figure 1A,B). Moreover, ha1-PLGA and ha2-PLGA showed a high degree of fiber alignment ranging mainly between −10° and + 10°. However, ha1-PLGA possessed a significantly higher percentage of fibers within these angles compared to ha2-PLGA ones (*p* < 0.05; Figure 1C), which can be attributed to the high polymer concentration resulted in more stable jets during the electrospinning process.

**Figure 2:**
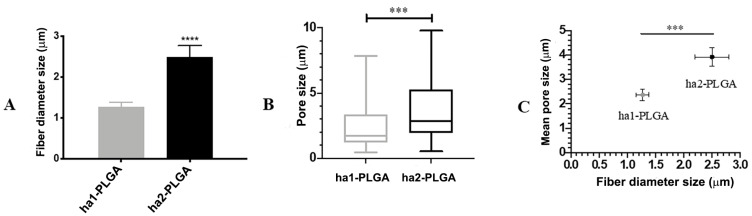
Fibers’ diameter and pore sizes of the ha1- and ha2-PLGA microfibers. (**A**) Histogram showing that ha1-PLGA microfibers have a diameter size of 1.27 ± 0.11 µm (ha1-PLGA) while ha2-PLGA 2.5 ± 0.27 µm. *** and **** Statistically significant values between ha1-PLGA and ha2-PLGA fleeces (*p* < 0.001 and *p* < 0.0001, respectively). (**B**) Frequency distribution of pore size within PLGA fleeces in which the mean pore sizes were 2.37 ± 0.24 μm for ha1-PLGA and 3.92 ± 0.39 μm for ha2-PLGA. (**C**) Comparison showing that increasing fiber diameter size of ha-PLGA fleeces is accompanied by a significant increase in pore size (*p* < 0.0001) (n = 3 for each type of fleece).

The fiber diameter sizes of the produced PLGA fleeces were significantly different and ranged between 1.27 ± 0.11 and 2.5 ± 0.27 µm for ha1-PLGA and ha2-PLGA, respectively (*p* < 0.0001; Figure 2A). In addition, by evaluating the mean pore size (Figure 2B), it can be seen that the PLGA fleeces with small fiber diameter (ha1-PLGA) possess a significantly smaller pore size (2.37 ± 0.24 μm) in respect to those with larger fiber diameter (ha2-PLGA; 3.92 ± 0.39 μm, *p* < 0.0001). Indeed, by comparing the mean of fiber diameter size with that of pore size, it can be noticed that increasing fiber diameter size increases significantly in turn the pore size (*p* < 0.0001; Figure 2C).

**Figure 3:**
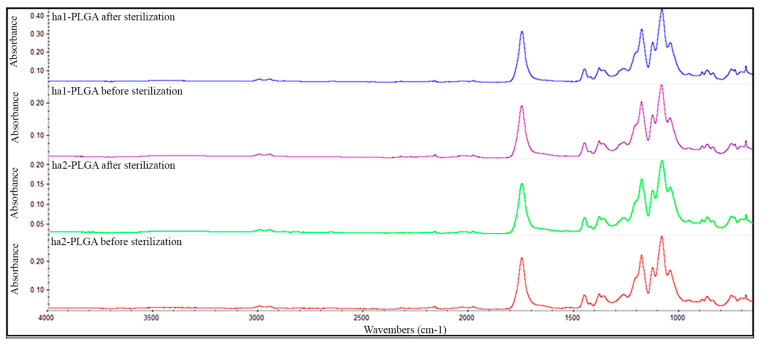
Representative FTIR spectra of ha1- and ha2-PLGA fleeces before and after sterilization. The spectra show no difference in the chemical composition of neat ha1- and ha2-PLGA fleeces before and after sterilization. The functional groups characterizing the PLGA as follows: ester carbonyl stretch (C=O) at 1748 cm^−1^, ether group stretch (C-O-C) at 1085 cm^−1^, and methyl stretching (C–H) and (C–CH_3_) at 1452 and 1044 cm^−1^, respectively.

FTIR technique was employed to determine if the sterilization step affected altering the functional groups of both types of the PLGA fleeces. As results, no differences have been detected neither in the shape nor in the position of the absorption peaks according to the FTIR spectra of the tested samples ha1- and ha2-PLGA fleeces before and after sterilization both in the shape and in the position of the absorption peaks (Figure 3). In detail, as shown in Figure 3, the samples exhibit a strong absorption band at wavenumber 1748 cm^-1^ due to the ester carbonyl stretch (C=O), stretching vibrations at 1085 cm^−1^ due to ether group (C–O–C) and two stretching vibrations at wavenumbers 1452 and 1044 cm^−1^ due to methyl stretching (C–H) and (C–CH_3_) groups, respectively, characterizing the PLGA material before sterilization. However, no changes in terms of switch or modification of the wavenumbers have been detected in the spectra of the analyzed samples after sterilization, confirming that the conducted sterilization process did not alter the raw chemical composition of the PLGA materials.

**Figure 4:**
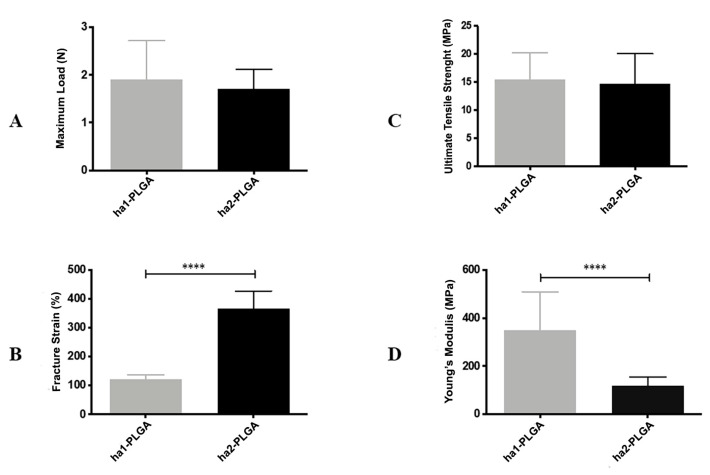
Mechanical characteristics of ha1- and ha2-PLGA fleeces. (**A**) Maximum Load (N); (**B**) Fracture Strain (%); (**C**) Ultimate Tensile Strength (MPa); (**D**) Young’s Modulus (MPa) carried out on ha1-PLGA and ha2-PLGA fleeces (n = 5 for each type of fleece, fleece dimension: 50 mm × 15 mm). Electrospun ha2-PLGA fleeces possess higher Fracture Strain values compared to those for ha1-PLGA, whereas ha1-PLGA fleeces possess higher Young’s Modulus values compared to those for ha2-PLGA. **** Statistically significant values between ha1-PLGA and ha2-PLGA fleeces (*p* < 0.0001).

Finally, Maximum Load, Ultimate Tensile Strength, Fracture Strain, and Young’s Modulus were measured to evaluate the effect of fiber diameter size on tensile properties of electrospun PLGA fleeces (Figure 4). Both electrospun ha1-PLGA and ha2-PLGA fleeces showed similar results in terms of the Maximum Load (1.90 ± 0.81N for ha1-PLGA and 1.71 ± 0.41 N for ha2-PLGA; *p* > 0.05) and the Ultimate Tensile Strength (15.52 ± 4.69 MPa for ha1-PLGA and 14.73 ± 5.29 MPa for ha2-PLGA; *p* > 0.05). Besides, the Young’s Modulus was significantly higher in the PLGA fleeces with small fiber diameter size (ha1-PLGA 350.3 ± 159.7 MPa vs. 117.7 ± 36.92 MPa ha2-PLGA; *p* < 0.0001), whereas the fracture strain was significantly more evident in PLGA fleeces with large fiber diameter size (ha2-PLGA 365.5 ± 62.06% vs. 122.4 ± 14.71% in ha1-PLGA; *p* < 0.0001).

### 3.2. AECs Seeding Efficiency and Proliferation on PLGA Fleeces

**Figure 5:**
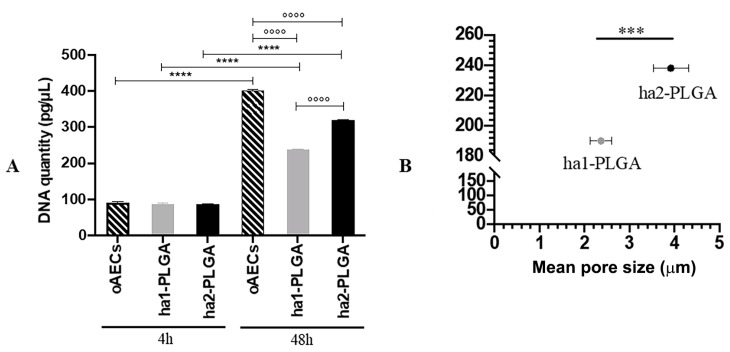
Cell seeding efficiency and the effect of fiber diameter size on oAECs’ proliferation. Cells were seeded on Petri dishes (oAECs) or ha1-PLGA and ha2-PLGA electrospun fleeces and allowed to grow for up to 48 h. (**A**) DNA quantification by Qubit^®^ dsDNA HS Assay on oAECs on Petri dishes or seeded fleeces after 4 h and 48 h of culture to verify cell adhesion, seeding efficiency, and to assess DNA quantity. It is shown that there is a higher proliferation rate in the case of ha2-PLGA compared to ha1-PLGA after 48 h of culture. *** and **** Statistically significant in the same sample type at different time points (*p* < 0.001 and *p* < 0.0001, respectively). ^°°°°^ Statistically significant between different sample types at 48 h (*p* < 0.0001) (n = 3 for each type of sample/analysis/time point, fleece size: 15 mm × 7 mm). (**B**) Comparison showing that increasing pore size of ha-PLGA fleeces is accompanied by an increase in cell proliferation rate confirmed by a significant increase of DNA quantity in ha2-PLGA compared to ha1-PLGA (*p* < 0.0001) after 48 h of culture.

DNA concentrations obtained from 0.05 × 10^6^ oAECs before seeding and after seeding onto both PLGA fleeces or on Petri dishes at 4 h and 48 h culture were quantified. The results clearly show that DNA concentration from oAECs before seeding corresponds to 91.66 ± 3.33 pg/µL (data not shown) and was comparable to those obtained after 4 h of cell seeding on Petri dish (90.94 ± 2.34 pg/µL; *p* > 0.05) and onto PLGA fleeces under static conditions (87.54 ± 1.75 pg/µL for ha1-PLGA vs. 86.52 ± 1.55 for ha2-PLGA; *p* > 0.05). These results confirm a high seeding efficiency onto both surfaces of PLGA fleeces and show also that the same number of cells adhered to fleeces independently to the fiber diameter size. Moreover, oAECs cultivated on Petri dish after 48 h showed a high DNA concentration (oAECs 48 h: 402.86 ± 1.06 pg/μL), whereas cells cultured on both types of fleeces proliferated less at this time period showing a significant decrease in DNA concentration (ha1-PLGA: 238 ± 1.67 pg/μL and ha2-PLGA: 320.46 ± 1.17 pg/μL, respectively, *p* < 0.05 vs. oAECs 48 h; Figure 5A). However, DNA quantity was higher in both fleeces after 48 h of culture compared to those detected after 4 h (*p* < 0.05; Figure 5A). In addition, DNA concentration was significantly higher in oAECs seeded on ha2-PLGA (320.46 ± 1.17 pg/μL) compared to those observed on ha1-PLGA (238 ± 1.67 pg/μL: *p* < 0.05; Figure 5A). By comparing DNA quantity and pore size (Figure 5B) it is evident that cell proliferation is highly dependent on pore size (*p* < 0.0001).

### 3.3. AECs viability, Distribution, and Morphology Within The Highly Aligned PLGA Fleeces

**Figure 6:**
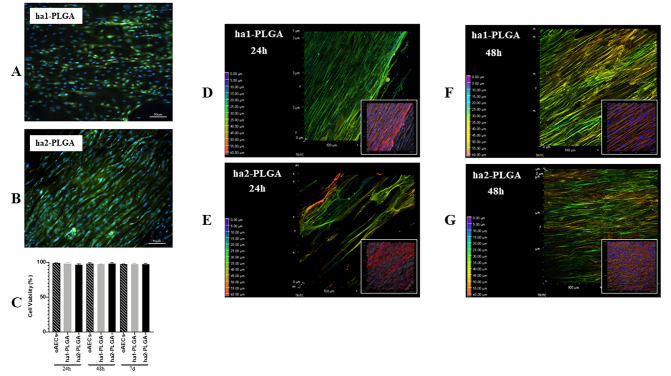
Amniotic Epithelial Stem Cell (AEC) viability and distribution on ha1- and ha2-PLGA fleeces. (**A**,**B**) Representative images assessing oAECs survival after 7 days culture on both types of fleeces with Calcein AM/propidium iodide (PI) stains (green and red fluorescence, respectively) where only viable cells are visible in green. Nuclei were counterstained with Hoechst 3342 (blue fluorescence). Scale bars = 50 μm (**C**) Histogram showing oAECs viability of cells seeded on ha1-PLGA or ha2-PLGA fleeces after 24 h, 48 h, and 7 days culture. No statistical difference was evident among the three groups (*p* > 0.05); (n = 3 for each type of fleece/time point, fleece size: 15 mm × 7 mm). (**D**–**G**) Representative XY confocal images showing oAECs distribution within ha1-PLGA and ha2-PLGA fleeces after 24 h and 48 h culture. The depth coded MaxIP analysis was used to assess cell penetration within the fleeces, visible as light background, by defining the gradient color related to the direction of the cells. The purple color of the gradient scale refers to the surface of the fleece, while the red color is attributed to the bottom of the surface. Thus, the most superficial cytoplasm cells are shown with green color, whereas the cytoplasm of the cells present at the bottom of the fleece are shown in red. It also is evident that oAECs have an optimal distribution within the fleeces, especially in ha2-PLGA after 24 h culture, while no differences were recorded between ha1- and ha2-PLGA fleeces in cell penetration after 48 h culture. The insets show the same phalloidin marked cell microphotographs that were analyzed with the MaxIP. In these images is evident the fusiform morphology of the cells attached to the aligned fibers. Fleeces captured from bright field exposition show that cells were disposed of parallelly oriented to the fibers. Scale bar = 50 µm.

AEC viability was confirmed by seeding cells for 24 h, 48 h, and 7 days onto ha1-PLGA or ha2-PLGA fleeces and then assessed by using Calcein AM (alive cells marker, green fluorescence) and Propidium Iodide stain (dead cells marker, nuclear red fluorescence). As shown in Figure 6A,B, after 24 h, 48 h, and 7 days of culture onto both types of PLGA fleeces, green fluorescent alive cells were evident (~ 98%; *p* > 0.05; Figure 6C) showing that PLGA is biocompatible for oAECs and allows cell integration and distribution within the fleeces (Figure 6A,B). Red nuclei from dead cells were found only very rarely (data not shown). 

Moreover, cells were stained with phalloidin (red fluorescence), an actin stain, to verify their cell morphology and penetration within the fleeces. 

The gradient of cell infiltration within the examined fleeces was carried out on phalloidin stained cells with the depth coded MaxIP analysis on the Z-stacks. This analysis automatically defines the gradient color related to the direction of the cytoplasm of cells within the fleece (in purple the surface, whereas in red the bottom of the fleece). It was possible to demonstrate an optimal cell migration and penetration within the fleeces, even if in ha2-PLGA the cells penetrated more in depth already after 24 h culture compared to those on ha1-PLGA (Figure 6D,E), whereas after 48 h of culture the infiltration degree was similar in both types of fleeces (Figure 6F,G).

**Figure 7:**
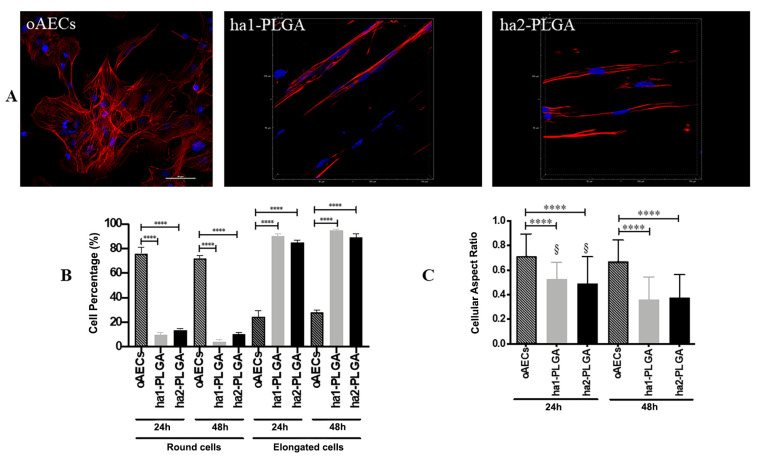
Morphology of oAECs on ha1-PLGA and ha2-PLGA fleeces. (**A**) Representative confocal images of oAECs morphology evidenced with phalloidin stain (red fluorescence) and nuclei counterstained with DAPI (blue fluorescence). It is evident that oAECs on Petri dishes maintain their typical epithelial shape, whereas they acquire a tenocyte-like elongated morphology already at 24 h culture on both ha1- and ha2-PLGA fleeces. Scale bar = 50 µm. (**B**) Percentage of the elongated oAECs after 24 h and 48 h seeding on ha-PLGA fleeces. **** Statistically significant values between the different studied groups at each time point (*p* < 0.0001). (**C**) Percentage of the cell nuclei aspect ratio after 24 h and 48 h seeding on ha1-PLGA and ha2-PLGA fleeces. **** Statistically significant values between the different studied groups at each time point (*p* < 0.0001). ^§^ Statistically significant values within the same group at different time points (*p* < 0.0001) (n = 3 for each type of fleece/analysis, fleece size: 15 mm × 7 mm).

Phalloidin stain allowed also to verify the morphology of the oAECs when cultured onto electrospun ha1- and ha2-PLGA fleeces for 24 h and 48 h. Actin staining with phalloidin (red fluorescence) shows that the cells were highly affected by the aligned topography in their morphology and alignment of the actin fibers. In particular, oAECs seeded on Petri dishes retained their typical epithelial cuboidal morphology and ovoid nuclei (Figure 7A). Differently, onto both types of ha1-PLGA and ha2-PLGA fleeces, oAECs acquired tenocyte-like elongated morphology (Figure 7B,C). On these samples, cell nuclei appeared fusiform (blue fluorescence), and parallelly oriented along the long axis of the fibrous PLGA matrix (Figure 6), further confirming the effect of fiber alignment guidance on cell morphology.

The percentage of elongated cells was then calculated after 24 and 48 h culture onto both types on ha-PLGA fleeces. After just 24 h culture, the percentage of oAECs with an elongated morphology was similar in both ha1-PLGA and ha2-PLGA fleeces (*p* > 0.05) but significantly higher with respect to cells on Petri dishes used as control (*p* < 0.0001) (90%, 85%, and 24%, respectively; Figure 7B). The number of elongated cells increased after 48 h of culture up to 95% and 89% in ha1-PLGA and in ha2-PLGA fleeces, respectively (*p* > 0.05) demonstrating that fiber alignment was able to transform oAECs from a cuboidal phenotype to an elongated morphology. Moreover, the cell nuclei aspect ratio, representing the ratio between nuclei width and its length, demonstrated that the cell nuclei onto both PLGA fleeces become more elongated after 48 h culture compared to 24 h culture (*p* < 0.0001; Figure 6C). This ratio was higher in oAECs seeded on Petri dishes, used as a control, (0.71 ± 0.18) confirming the ovoidal shape nuclei (i.e., 1 is perfectly circular) compared to the ratios obtained with the cells seeded onto both electrospun PLGA fibers relative to after 24 h that were significantly lower (0.53 ± 0.14 for ha1-PLGA and 0.49 ± 0.22 for ha2-PLGA with respect to 0.71 ± 0.18 for oAECs; *p* < 0.0001). Additionally, the cellular aspect ratio decreased further after 48 h culture in the cells seeded onto ha1-PLGA (0.36 ± 0.18) and ha2-PLGA (0.37 ± 0.19) with respect to 0.67 ± 0.17 for oAECs (*p* < 0.0001). However, by comparing the cellular aspect ratios of the cells seeded onto the two PLGA fleeces types, it was possible to verify a significant decrease in this ratio between the two culture time points (0.53 ± 0.14 vs. 0.36 ± 0.18 for ha1-PLGA and 0.49 ± 0.22 vs. 0.37 ± 0.17 for ha2-PLGA, *p* < 0.0001) demonstrating that increasing the cell culture time onto both types of ha-PLGA fleeces lead to a further elongation in cell morphology.

### 3.4. Fiber Diameter of ha-PLGA Fleeces Induce an Early oAECs’ Tenogenic Differentiation

Considering the tenocyte-like morphology acquired by the oAECs seeded onto the both PLGA fleeces, it was verified their teno-inductive potential. In particular, it was verified if the fiber diameter size affected the tenogenic potential on AECs without adding tenogenic supplementation in the culture media.

**Figure 8:**
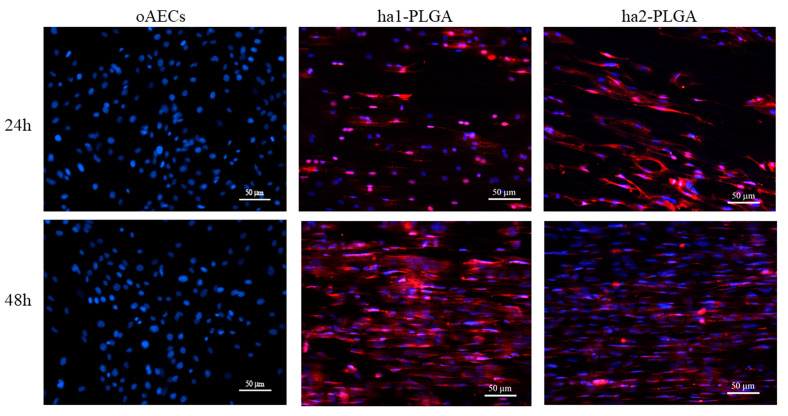
Teno-inductive properties of ha1- and ha2-PLGA fleeces on AECs. IHC analysis revealed COL1 protein expression (red fluorescence) in oAECs when seeded onto ha1-PLGA and ha2-PLGA; whereas cells cultured on Petri dishes do not express the protein. DAPI (blue fluorescence) counterstained nuclei. Images show that cells seeded onto Petri dishes never express COL1, whereas it is expressed in AECs cultured on both ha1- and h2-PLGA fleeces already after 24 h culture. Positivity to COL1 was evident in the cytoplasm of the oAECs with a tenocyte-like morphology. Scale bars = 50 μm (n = 3 for each type of sample/ time point, fleece size: 15 mm × 7 mm).

AECs’ tenogenic differentiation was firstly confirmed by analyzing COL1 protein, the most abundant protein in tendons, by using the immunofluorescence technique on oAECs seeded on Petri dishes, and onto ha1- and ha2-PLGA fleeces after 24 h and 48 h of culture. oAECs, normally do not express COL1 [34], and as shown in Figure 8 they were still negative to this protein when cultured on Petri dishes for 24 h and 48 h of culture. Instead, COL1 was already expressed after just 24 h culture onto both PLGA fleece types (Figure 8) and positivity was maintained at 48 h culture (Figure 8).

### 3.5. Fiber Diameter of ha-PLGA Fleeces Induce an Early oAECs’ Tenogenic Differentiation

**Figure 9:**
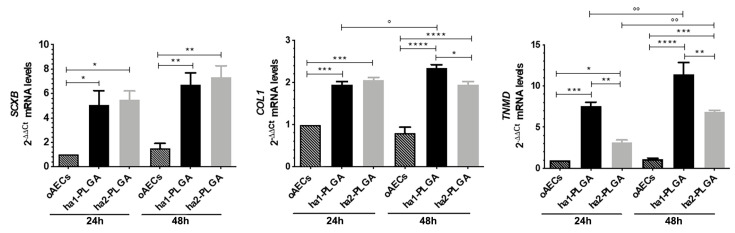
Teno-inductive properties of ha-PLGA fleeces on AECs. SCX, COL1, and TNMD gene expression were evaluated at 24 h and 48 h culture points. Quantitative RT-PCR data show a significantly higher expression of tenogenic markers in oAECs seeded onto ha-PLGA fleeces compared to oAECs on Petri dishes (reference sample oAECs at 24 h = 1) (n = 3 for each type of sample/analysis/time point, fleece size: 15 mm × 7 mm). * Statistically significant among the different groups at each time point (*p* < 0.05). ** Statistically significant among the different groups at each time point (*p* < 0.01). *** Statistically significant among the different groups at each time point (*p* < 0.001). **** Statistically significant among the different groups at each time point (*p* < 0.0001). ° Statistically significant within the same group at different time points (*p* < 0.05). °° Statistically significant within the same group at different time points (*p* < 0.01).

To better understand in depth oAECs tenogenic differentiation, it was analyzed the gene expression profile of three tendon-related genes: *SCX* (early tenogenic marker), *COL1* and *TNMD* (mature tendon markers). In particular, *SCX* and *COL1* gene expression were significantly up-regulated in oAECs seeded onto both ha-PLGA fleeces with respect to oAECs after 24 h culture (*p* < 0.05; Figure 9). *TNMD* gene expression was significantly higher after 24 h culture with respect to oAECs in cells cultured onto both ha-PLGA fleeces, even if the significantly higher expression was noticed in ha1-PLGA with respect to ha2-PLGA (*p* < 0.001 for ha1-PLGA and *p* < 0.05 for ha2-PLGA). Moreover, it increased significantly with respect to oAECs in both types of ha-PLGA fleeces after 48 h culture (*p* < 0.0001 for ha1-PLGA and *p* < 0.001 for ha2-PLGA; Figure 9). After 24 h culture, *SCX* and *COL1* mRNA gene expression were similarly up-regulated in ha1- and ha2-PLGA fleeces (*p* > 0.05; Figure 9), whereas *TNMD* mRNA gene expression was differently expressed in ha1-PLGA vs. ha2-PLGA (*p* < 0.01). All tendon-related gene expression significantly increased after 48 h (*p* < 0.01; Figure 9). In detail, *SCX* was more upregulated in ha2-PLGA fleeces with respect to ha1-PLGA (*p* > 0.05), while conversely *COL1* and *TNMD* were significantly more upregulated in ha1-PLGA fleeces with respect to ha2-PLGA (*p* < 0.05 and *p* < 0.01 for *COL1* and *TNMD* mRNA gene expression, respectively; Figure 9).

### 3.6. Fiber Diameter of ha-PLGA Fleeces Differently Stimulates oAECs’ Immunomodulatory Potential

**Figure 10:**
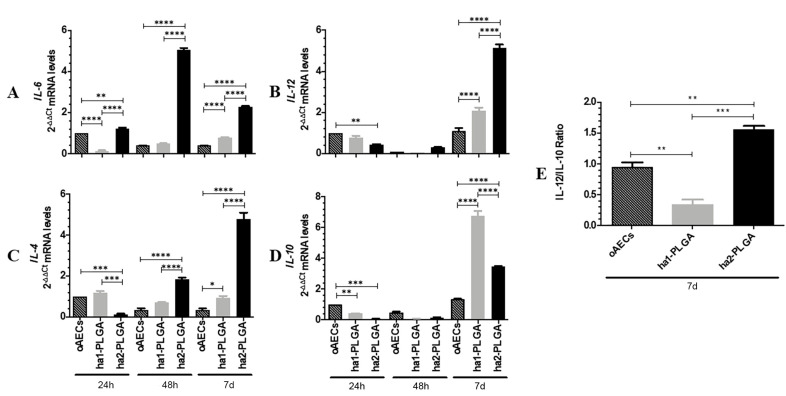
Anti-inflammatory and pro-inflammatory ILs expression profiles in oAECs seeded on ha1- and ha2-PLGA fleeces. (**A** and **B**) Pro-inflammatory IL-6 and IL-12 and (**C** and **D**)Anti-inflammatory IL-10 and IL-4 mRNA expressions were studied in oAECs and in oAECs seeded on ha1-PLGA and on ha2-PLGA fleeces after 24 h, 48 h and 7 days of culture. oAECs after 24 h of culture on Petri dish was used as the reference sample (oAECs at 24 h = 1). (**E**) IL-12/IL-10 ratio of the expression levels in oAECs and in oAECs seeded on ha1-PLGA and ha2-PLGA fleeces after 7 days (7 d) of culture. oAECs after 7 days of culture on Petri dish was used as the reference sample (oAECs at 7 d = 1). Relative quantification was done using the ΔΔCt method and GAPDH was used as the housekeeping gene. The data are mean ± SD of 3 replicates (n = 3 experimental replicates) performed in oAECs collected from at least 3 fetuses (n = 3 biological replicates). * Statistically significant among the different groups at each time point (*p* < 0.05). ** Statistically significant among the different groups at each time point (*p* < 0.01). *** Statistically significant among the different groups at each time point (*p* < 0.001). **** Statistically significant among the different groups at each time point (*p* < 0.0001).

To gain insights into the effect of fiber diameter size on the immunomodulatory properties of oAECs seeded onto ha1-PLGA and ha2-PLGA fleeces; the gene expression analysis of key anti-inflammatory (IL-4 and IL-10) and pro-inflammatory (IL-6 and IL-12) cytokines were performed after 24 h, 48 h and 7 days of culture. oAECs for the same time period of culture were considered as a control of the gene expression profile. As reported in Figure 10, cytokines’ expression profile was differently modulated in oAECs along with the culture time points depending on fiber diameter size.

oAECs expressed basal levels of the cytokines for the entire culture period (Figure 10A–D).

All analyzed ILs were expressed in basal levels in cells cultured onto ha1-PLGA fleeces similarly to oAECs at 24 h and 48 h of culture. However, these cells showed a significant increase in the expression of both pro- and anti-inflammatory cytokines at 7 days of culture (*p* < 0.05 vs. oAECs, Figure 10A–D). Interestingly, IL-10 was significantly higher in the cells seeded onto this fleece (*p* < 0.0001 vs. oAEC or ha2-PLGA).

On the contrary, in oAECs seeded on ha2-PLGA fleeces, the expression profiles of IL-6 and IL-4 significantly increased already starting from 24 h and 48 h, respectively (IL-6, *p* <0.01 vs. oAECs or ha1-PLGA, Figure 10A; IL-4, *p* < 0.001 vs. oAECs or ha1-PLGA, Figure 10C) and remained both at high levels after 7 days of culture (*p* < 0.0001 vs. oAECs or ha1-PLGA; Figure 10A). IL-12 expression instead, significantly increased in respect to both oAECs or ha1-PLGA after 7 days culture (*p* <0.0001 vs. oAECs or ha1-PLGA; Figure 10B) while that of IL-10 increased significantly in respect to oAECs (*p* < 0.0001) and remained significantly lower in respect to ha1-PLGA (*p* < 0.0001).

The analysis of IL-12/IL-10 ratio at 7 days culture indicates that it is significantly low in ha1-PLGA fleeces even when compared to the levels observed in oAECs (*p* < 0.01, ha1-PLGA vs. oAECs at 7 days; Figure 10E) suggesting that the smaller fiber diameter size induces anti-inflammatory properties to the seeded cells. However, the high IL12/IL10 ratio of ha2-PLGA fleeces (*p* < 0.01, ha2-PLGA vs. oAECs and vs. ha1-PLGA; Figure 10E) indicates that the larger fiber diameter size stimulates the expression of pro-inflammatory IL-12 and IL-6, despite the high production of IL-4 and IL-10.

## 4. Discussion

Tendon tissue engineering strategies expect to use scaffolds that mimic the structure and mechanical behavior of the tissue to stimulate cells towards regenerative profiles. In this study, electrospun PLGA fleeces with two different microfiber diameter size (1.27 ± 0.11 µm for ha1-PLGA and 2.5 ± 0.27 µm for ha2-PLGA) were fabricated to mimic the pattern of the tendon ECM, fiber alignment, and the diameter of collagen fibers of a healthy tendon. On fabricated fleeces, it was then analyzed the influence of fiber diameter size on the biological response of oAECs. 

These tendon biomimetic PLGA fleeces have been fabricated by electrospinning with an optimized rotating drum collector with a high rotational speed to fabricate reproducibly electrospun fibers. Electrospinning technique is well consolidated in the field of tendon tissue engineering since it allows to fabricate fibers with high surface area for cell attachment and high porosity to promote nutrient and waste exchange [69,70,71,72,73,74,75], by using different natural (collagen and fibrin [76]) and synthetic (PCL [53], PLLA [70], chitosan [52]) polymers. It is known that PLGA, in particular, provides sufficient control of degradation [77,78] conjugated to adequate mechanical strength that fosters its application in tissue remodeling and regeneration [79,80], and thus widely used in tendon tissue engineering. 

In this research, by varying the parameters of the electrospinning machine as polymer concentration, flow rate, voltage, distance between needle-collector, it was possible to specifically obtain highly aligned microfiber ha-PLGA fleeces with two different diameter size of 1.27 ± 0.11 µm (ha1-PLGA) and 2.5 ± 0.27 µm (ha2-PLGA), within the range also reported in other electrospun PLGA scaffolds for tendon tissue engineering (from less to over 1 µm in diameter) [46,49,59,81]. In addition, fiber diameter sizes used in this study were within the range of healthy tendons or ligaments fibers amongst which tenocytes reside (1–20 µm) [49,57,59,82]. In agreement with previous works [83,84], it was noticed that the increase of fiber diameter size corresponded to an increase in a linear way to the pore size of the fibers. On the other hand, fleeces with high PLGA concentration led to a better fiber alignment compared to those with a low PLGA concentration due to the increase in the solution viscosity rendering the jet coming from the Taylor cone more stable.

Furthermore, it was analyzed if the sterilization procedure, which is a critical step to be considered when using fleeces for biological applications, could alter PLGA bulk composition. This study confirmed that the sterilization procedure used on PLGA fleeces did not alter their chemical configuration and composition [51].

Both types of PLGA fleeces had mechanical properties values that are similar to those reported for tendons and ligaments, as human Achilles tendons (28–86 MPa) [85,86], human patellar tendons (5–65 MPa) [85,87], human rotator tendons (14–45 MPa), and human Achilles graft (16 MPa) [85], even if the fracture strain values were more similar to the electrospun fleeces with small fiber diameter size (ha1-PLGA) [85]. The maximum load and the ultimate tensile strength of both electrospun PLGA fleeces were similar, whereas fleeces with small fiber diameter (ha1-PLGA) showed remarkably high Young’s modulus but low breaking strain compared to those with larger fiber diameter (ha2-PLGA). It has been determined that decreasing fiber diameter size increases the Young’s modulus property of the fleeces, while decreasing that of elongation at break. Doubling the fiber diameter results in a significant increase of the fracture strain, which in the end leads to an increase of the load capacity when the fiber is stretched and thus to an increase of the elasticity (reduction of stiffness). Despite differences with other literature data [59,88] the variation in the results could be attributed to the polymer type itself as well as to the chemistry and the solution properties within the same polymer type. 

Interestingly, it has been suggested that electrospun scaffolds with fiber contained in the micron range (~1.40 µm to ~1.80 µm), such as those obtained for this research, rather than fibers in the nano range (~300 nm to ~650 nm) are of choice to maintain tenocytes’ phenotype [59,60]. Nano-fibers were not considered in this research since these would have a similar size of collagen fibrils and are also characteristic of the fibrotic tissue produced during the healing process [60,89]. In particular, it was suggested that while nanofibers recapitulate the early stages of tissue repair [49,60,77,90,91], micron-fibers mimic the healthy tendon collagen fibers during the tissue remodeling phase [49]. Moreover, microfiber scaffolds promote better cell teno-differentiation and higher cell alignment and elongation in comparison to nanofibrous scaffolds [49,60]. 

Importantly, in this research, it was demonstrated that both fiber alignment and diameter size regulate oAECs proliferation and penetration, while only fiber alignment influences cell morphology. In turn, the intrinsic physical cues of the tendon mimetic aligned PLGA fleeces were able to boost oAECs tenogenic differentiation and positively influenced anti-inflammatory/pro-healing gene expression. These biological responses were dependent on the fiber diameter, especially with the lower fiber diameter size (1.27 μm).

The positive outcome of engineered stem cell-scaffold depends on the scaffold’s biocompatibility for the seeded cells which are functionally stimulated by its physical signal cues. In tendon tissue engineering, many studies have used different types of cell sources, mainly mesenchymal ones of different origin such as fibroblasts [46,57,92,93], BMMSCs [94,95], ADMSCs [53,96,97,98], tenocytes [64], and TPSCs [11,13]. Nonetheless, these cells are already fusiform in their morphology and already express collagen type 1, thus making challenging the demonstration of the topological effect of the fibers on these cells’ differentiation. Instead, by using oAECs it is possible to specifically evaluate the biological performance and functionality of tendon biomimetic scaffolds. This cell source, indeed, allowed to in depth verify the topological effect of the fleeces and the mechanisms that permitted to an epithelial cell (cuboidal in its morphology and negative to collagen type 1 expression) to differentiate and be committed towards a mesenchymal cell of the tenogenic lineage [51]. In previous studies, it was already demonstrated oAECs’ biocompatibility for PLGA [50], the early teno-inductive effect of highly aligned PLGA fibers on cells through an EMT-mediated pathway [51]. Of note, oAECs conjugate high plasticity [33,99,100], with a great ability to produce growth factors sustaining key processes involved in tissue regeneration, such as immunomodulation of both innate and adaptive immune systems [101,102,103,104,105,106], and these properties allow them to be used in allo- and xeno-transplantation settings [33].

The polymer chemistry of these scaffolds is essential for cell adhesion, migration, proliferation, and differentiation that occur only after cells are securely adhered [107]. Even if the seeding efficiency was similar on both types of PLGA fleeces, after 48 h of culture, oAECs engineered with ha1-PLGA fibers showed a significant reduction of DNA quantity and a slower migration within the fleece compared to ha2-PLGA, which had both increased fiber diameter and pore size.

As supported by prior studies, the findings herein presented confirm that fiber alignment guides cell organization and morphology along with the fiber arrangement [51,52,53,60,70,108,109], without being affected by the fiber diameter size. Indeed, the aligned topology of PLGA fibers influenced oAECs morphology since these cells acquired an elongated tenocyte-like shape in opposition to the oAECs cultured on Petri dishes or on randomly oriented PLGA fiber fleeces, which retained a native cuboidal morphology [51]. It was interesting to notice that analyzing the cell aspect ratio, oAECs became more fusiform at 48 h of culture with respect to 24 h onto both ha1- and ha2-PLGA fleeces.

Moreover, by keeping fiber alignment constant, it was possible to decouple the effects based on fiber diameter. It was also demonstrated that not only fiber topography [51] but also fiber diameter size had a great influence on the teno-differentiation ability of oAECs. In Russo et al. [51] oAECS were cultured on the scaffolds up to 28 days, showing an upregulation of *COL1* and *TNMD* already after 48 h of culture. In this study fleeces’ teno-inductive effect was verified also before 48 h of culture. Interestingly, it was demonstrated that seeded oAECs teno-differentiated already after 24 h of culture. In particular, they acquired a tenocyte-like morphology, up-regulated tendon related genes (*SCX*, *COL1*, and *TNMD*), and the expressed COL1 protein. oAECs expressed COL1 protein in their cytoplasm independently to the fiber diameter size of both ha-PLGA fleeces. These results were very encouraging, as in other publications where MSCs were engineered onto aligned fibers, COL1 protein was upregulated after 3 [46,57] or 7 days [48] of culture. The influence of fiber diameter size on oAECs’ teno-differentiation was evident by in depth analyzing tendon-related gene expression profiles. *SCX* was more upregulated in ha2-PLGA fleeces, whereas *COL1* and *TNMD* were more upregulated in ha1-PLGA fleeces. This result is in accordance with other works that confirmed the influence of fiber diameter on tenogenic differentiation of MSCs occurred after at least 3 days of culture [46,49,59,76,98]. Even if the expression profile of *SCX* at 48 h culture resulted higher in ha2-PLGA fleeces, cells cultured onto ha1-PLGA fleeces express higher levels of the downstream TNMD, suggesting an earlier and/or more efficient activation of SCX under this condition, which is indicative of a more sustained commitment of the oAECs toward the tenogenic lineage [110,111]. Thus, ha1-PLGA fleeces tend to increase the gene expression of the markers considered crucial for inducing the tenogenic commitment of oAECs and regulating tenocyte phenotype. This is an interesting data if it is considered that SCX is an early marker involved in tendon formation during fetal life, whereas in adult tendons, its basal expression suggests a positive role in homeostasis as co-activator of other tendon-related genes, such as COL1 and TNMD. Indeed, TNMD is essential to ensure the collagen network formation and tendon organization, whereas COL1 is the most abundant protein in a native tendon [112]. The observed response of oAECs on the aligned microfibers also confirms the previous findings exploring human tendon fibroblast response to aligned PLGA microfibers [59].

These observations suggest that fiber alignment is essential in directing cell adhesion and organization. Instead, fiber diameter maintains tenocyte-like phenotype. Both parameters influence the cells towards physiological healing. Thus, aligned ha1-PLGA (~1.27 μm) rather than ha2-PLGA (~2.5 μm) fleeces are an optimal matrix for tendon regeneration when oAECs are used as a cell source for tendon tissue engineering.

Scaffolds can also contribute to positively modulate the inflammatory response of the native tissue and stimulate its regeneration. Although, when implanted, these could induce inflammation in the host tissue compromising the regeneration if this process is not controlled [112,113,114]. Immunomodulation through biomaterials, alignment of the scaffolds, and fiber diameter affects the polarization of macrophages from inflammatory (M1) to anti-inflammatory/pro-regenerative (M2) phenotypes [61,63,113,114] or even potentiates stem cell immunomodulatory function [62,98,115]. In particular, it has been demonstrated that aligned fibrous matrices alone facilitate the phenotypic switch of macrophages from M1 to M2 by modifying their morphology [98,114,116]. Fiber diameter size has shown effects on promoting macrophage elongation, reducing pro-inflammatory cytokine production, and priming these cells for differentiation into M2 macrophages [63,114,117,118]. It has been demonstrated both in in vitro and in vivo settings that AECs possess immunomodulatory properties playing a fundamental paracrine role in regenerative medicine [25,34,35]. In fact, amongst AECs immunomodulatory properties there is the suppression of stimulated T cell proliferation and the modulation of macrophage polarization [119,120]. In particular, independently from the gestational stage of origin, oAECs downregulate lymphocytes proliferation and inhibit PBMCS in in vitro culture [119]. Furthermore, it has been demonstrated that AECs transplanted into disease models of lung and liver fibrosis contrast the inflammatory and fibrotic response [105]. Preclinical evidence has shown that oAECs were able to enhance tendon healing via paracrine-mediated inhibition of inflammatory immune cells, including monocyte/macrophages, T cells, and lymphocytes [29,34,35,121] with a prompt reduction of pro-inflammatory cytokines (IL-12) and pro-fibrotic cytokines/growth factors and the rapid enhancement of pro-regenerative cytokines (IL-10) [29,33,103]. In particular, oAECs during in vivo tendon regeneration prevent monocytes activation towards dendritic cells and promote the shift from inflammatory M1 to anti-inflammatory M2 macrophage subpopulations [29]. The therapeutic efficacy of AECs secretomes has even been confirmed on sportive horses affected by spontaneous SDFT tendinopathy where they have accelerated the recovered sportive performance and significantly reduced the rate of recurrences [32].

To our knowledge, no studies have explored yet the effects of PLGA fiber alignment and fiber diameter size on the immunomodulation capabilities of oAECs. Thus, in this research, the gene expressions related to the immunomodulatory secretome of oAECs cultured onto ha1- and ha2-PLGA fleeces were also investigated. The obtained results demonstrate that PLGA aligned fibers were able to promote the production of immunomodulatory factors in oAECs, which is essential for dictating favorable host responses and is beneficial for tissue repair. In particular, the expression of both pro (IL-12 and IL-6) and anti-inflammatory markers (IL-4 and IL-10) [29,35,98] were analyzed after 24 h, 48 h, and 7 days of culture on oAECs seeded on the two ha-PLGA constructs. It was interesting to verify that fiber diameter size greatly influenced ILs’ expression in oAECs. In detail, cells cultured onto ha2-PLGA fleeces (~2.5 µm) showed an upregulation of both anti-inflammatory and pro-inflammatory markers. Instead, ha1-PLGA fleeces were also able to increase the expressions of anti-inflammatory cytokines IL-4 and IL-10 after 7 days of culture, especially for IL-10. Relevantly, ha1-PLGA fleeces showed a lower IL-12/IL-10 ratio indicating that probably if transplanted in vivo, the bio-hybrids with the smaller fiber diameter size (ha1-PLGA) could better stimulate an Mφ skewing towards M2 phenotype. Indeed, the low IL-12/IL-10 ratio, accordingly to Manuelpillai et al. [105] and Mauro et al. [29] observations in hAECs xeno-transplanted liver and in oAECs allo-transplanted tendons, respectively, seems to support a rapid transition of AECs from the inflammatory to the reparative phase indicating an Mφ skewing towards M2 phenotype. Of note, the amount and length of the inflammatory process may depend on varying the morphological and chemical properties of the scaffold. Consequently, it may be important to evaluate cell responses in the early (24 h) and late inflammation (7 days) after biomaterial transplantation [62]. In addition, the healing process of an injured tendon has an initial inflammatory phase, where in the first 24 h inflammatory cells such as neutrophils, monocytes, and macrophages are attracted to the injury site by pro-inflammatory cytokines and this process lasts few days (about 7 days) [8]. Thus, it is crucial to understand how to modulate this phase allowing direct tendon healing towards regeneration and not fibrosis. However, before in vivo preclinical trials can be conducted, it is essential to collect robust in vitro evidence as fleece biomimicry, teno-inductive potential, and immune-induction. The obtained results suggest that the effects given from an aligned topography combined with the smaller fiber diameter (ha1-PLGA) have positively influenced oAECs’ teno-differentiation and immunomodulatory behavior. Indeed, these results suggest that these biohybrid fleeces with small fiber diameter size might potentially promote subsequent in vivo tissue regeneration also through immunomodulatory factors released from the seeded stem cells that would be stimulated to produce pro-regenerative/anti-inflammatory cytokines for long time. 

The role of scaffold’s topography together with its fiber diameter size in modulating the implant-tissue reaction is becoming increasingly important. Thus, the design of aligned PLGA fleeces with 1.27 µm fiber diameter size could be considered simultaneously as immune-modulatory and teno-regenerative materials that could tune the in vivo regenerative response. The ha1-PLGA fleeces or their upgrade into 3D scaffolds could be applied in tendon tissue engineering both for domestic animal patients (i.e., horse and dogs) and humans, which are highly affected by tendinopathies, in a “one health medicine” vision.

## 5. Conclusions

This is the first research, to our knowledge, that explores the effect of scaffolds’ fiber diameter size on oAECs’ teno-differentiation and their immunomodulatory properties.

Overall, the reported results support the use of the proposed aligned PLGA microfiber fleece, especially with the smaller fiber size (ha1-PLGA: 1.27 µm) in tendon tissue engineering, since they can synergistically boost oAECs tenogenesis and modulate their anti-inflammatory/pro-healing paracrine signaling, thus collectively contributing to improving the regenerative potential of tendon ruptures. Therefore, even if cell function is controlled by soluble factors, matrix design gives equally important cues in modulating oAECs’ biology. These biological mechanisms that are starting to be clarified on the effect of fiber alignment and diameter size of electrospun PLGA fleeces on AECs’ biology, give important information that could pave the way to in vivo studies. This could increase their clinical efficacy particularly for inflammatory/immune-related diseases as tendinopathies. Preclinical studies for tendon injury using relevant animal models (i.e, sheep) are necessary to quantitatively evaluate oAECs-biohybrid ha1-PLGA fleece’s regenerative potential. Altogether, all these biological positive responses could reduce tissue fibrosis at the injury site and improve tendon engineering strategies.

## Figures and Tables

**Table 1:** Electrospinning parameters.

	Ha1-PLGA	Ha2-PLGA
PLGA concentration (%*wt*/*wt*)	8	12
Flow rate (mL/h)	1.75	0.25
Applied voltage (kV)	26	33
Needle-collector distance (cm)	20	20
Relative humidity (%)	34.5	60
Temperature (°C)	21.5	22.5

## Supplementary Materials

The following are available online at https://www.mdpi.com/2073-4409/9/5/1207/s1, Table S1: Sequences of primers and conditions used in Real-Time qPCR.
