# Supplementary material for: Electrospun PLGA Fiber Diameter and Alignment of Tendon Biomimetic Fleece Potentiate Tenogenic Differentiation and Immunomodulatory Function of Amniotic Epithelial Stem Cells
Source: Cells. 2020 May 13;9(5):1207. doi: 10.3390/cells9051207 (PMC7290802; doi:10.3390/cells9051207)
Supplement: Supplementary file 1 [file cells-09-01207-s001.pdf]

Table S1

Table S1. Sequences of primers and conditions used in Real-Time qPCR.

Gene	Primer sequences	Annealing Tm (°C)
COL1[35]	F: 5′-CGTGATCTGCGACGAACTTAA-3′<br>R: 5′-GTCCAGGAAGTCCAGGTTGT-3′	62
TNMD[35]	F: 5′-TGGTGAAGACCTTCACTTTCC-3′<br>R: 5′-TTAAACCCTCCCCAGCATGC-3′	62
SCXB[35]	F: 5′-AACAGCGTGAACACGGCTTTC-3′<br>R: 5′-TTTCTCTGGTTGCTGAGGCAG-3′	64
IL-6[67]	F: 5′-ACCTGGACTTCCTCCAGAAC-3′<br>R: 5′-TTGAGGACTGCATCTTCTCC-3′	62
IL-4[67]	F: 5′-AAGCCCTCAGCTAAGCATGT-3′<br>R: 5′-AGGCATCACAGGCTCAAGTC-3′	63.2
IL-10[67]	F:5′-CCAGGATGGTGACTCGACTAG-3′<br>R: 5′-TGGCTCTGCTCTCCCAGAAC-3′	65.3
IL-12b[67]	F: 5′-TCAAACCAGACCCACCCAAG-3′<br>R: 5′-CACAGATGCCCATTCACTCC-3′	65.6
GAPDH[67]	F: 5′-TCGGAGTGAACGGATTTGGC-3′<br>R: 5′-CCGTTCTCTGCCTTGACTGT-3′	64.4
